# Enzyme-Specific Activation versus Leaving Group Ability

**DOI:** 10.1002/cbic.201200227

**Published:** 2012-07-23

**Authors:** Roseri J A C de Beer, Berry Bögels, Gijs Schaftenaar, Barbara Zarzycka, Peter J L M Quaedflieg, Floris L van Delft, Sander B Nabuurs, Floris P J T Rutjes

**Affiliations:** a Institute for Molecules and Materials, Radboud University NijmegenHeyendaalseweg 135, 6525 AJ Nijmegen (The Netherlands); b Computational Drug Discovery, Center for Molecular and Biomolecular Informatics, Radboud University Nijmegen Medical CentreP. O. Box 9101, 6500 HB Nijmegen (The Netherlands); c DSM Innovative Synthesis BVP. O. Box 18, 6160 MD Geleen (The Netherlands)

**Keywords:** enzyme catalysis, enzyme-specific activation, guanidinophenyl ester, papain, peptides

## Abstract

Enzyme-specific activation and the substrate mimetics strategy are effective ways to circumvent the limited substrate recognition often encountered in protease-catalyzed peptide synthesis. A key structural element in both approaches is the guanidinophenyl (OGp) ester, which enables important interactions for affinity and recognition by the enzyme—at least, this is usually the explanation given for its successful application. In this study we show that leaving group ability is of equal or even greater importance. To this end we used both experimental and computational methods: 1) synthesis of close analogues of OGp, and their evaluation in a dipeptide synthesis assay with trypsin, 2) molecular docking studies to provide insights into the binding mode, and 3) ab initio calculations to evaluate their electronic properties.

## Introduction

These days enzymes are commonly used in organic synthesis.[^1^] The benefits of enzymatic reactions, including their generally excellent regio- and enantioselectivity, are widely recognized. Because of the usually mild reaction conditions, enzymatic conversions are often regarded as a green alternative to classic organic reactions. However, the limited substrate scope of many enzymes remains a big disadvantage.

One of the areas of application of enzymes is peptide synthesis. In this field, proteases are employed to form the peptide bonds (which they would natively hydrolyze) by exploiting the reversibility of chemical reactions. A prerequisite for this enzymatic activity, irrespective of whether aqueous media or organic solvents are used, is that specific amino acids are recognized.[^2^] This problem of recognition can be circumvented by applying the “substrate mimetics” strategy as previously described for trypsin and other proteases.[^3^] The guanidinophenyl (OGp) ester, which in essence resembles the naturally recognized side chain of arginine, is claimed to serve as a recognition moiety for trypsin, thereby making recognition independent of the side chain of the amino acid and thus broadening substrate scope. This approach is typically applied under aqueous conditions; but because OGp also functions as a leaving group, the commonly occurring secondary hydrolysis is prevented, as the product formed becomes unrecognizable for the enzyme.

A similar solution to limited substrate acceptance was found for papain, that is, to overcome enzyme-specific activation.[^4^] Based on docking studies, the OGp group is predicted to bind to papain in a different orientation than the natural substrate, arginine. By taking advantage of this alternative recognition, papain is able to catalyze dipeptide formation without being restricted to specific amino acid residues. We noticed these versatile applications of the OGp moiety and wondered what was the reason for these remarkable properties. Additional research, in which OGp was replaced with simpler esters, indicated that besides enzyme recognition and affinity, the leaving group ability may be an important factor.[^5^] In this study, the contribution of these components was investigated by both experimental and computational methods. Several analogues of OGp were designed, synthesized, and docked to trypsin to provide insight in the binding mode. Subsequently, their effectiveness in dipeptide formation was experimentally determined, and an attempt was made to increase the activity by further variation of one of the analogues. An ab initio study provided insight into the electronic properties of the analogues under investigation.

## Results and Discussion

### Prediction of the binding mode of OGp analogues to trypsin

To distinguish between the effects of affinity for the enzyme and leaving group ability, we evaluated a set of close analogues of OGp that differed slightly in both properties. We opted for trypsin as the model system, because this enzyme is highly specific for arginine, in contrast to papain, which exhibits broad substrate specificity, with only a slight preference for arginine. Moreover, the catalytic mechanism of the serine protease trypsin,[^6^] also with respect to substrate mimetics,[^7^] is well known.

**Scheme 1:**
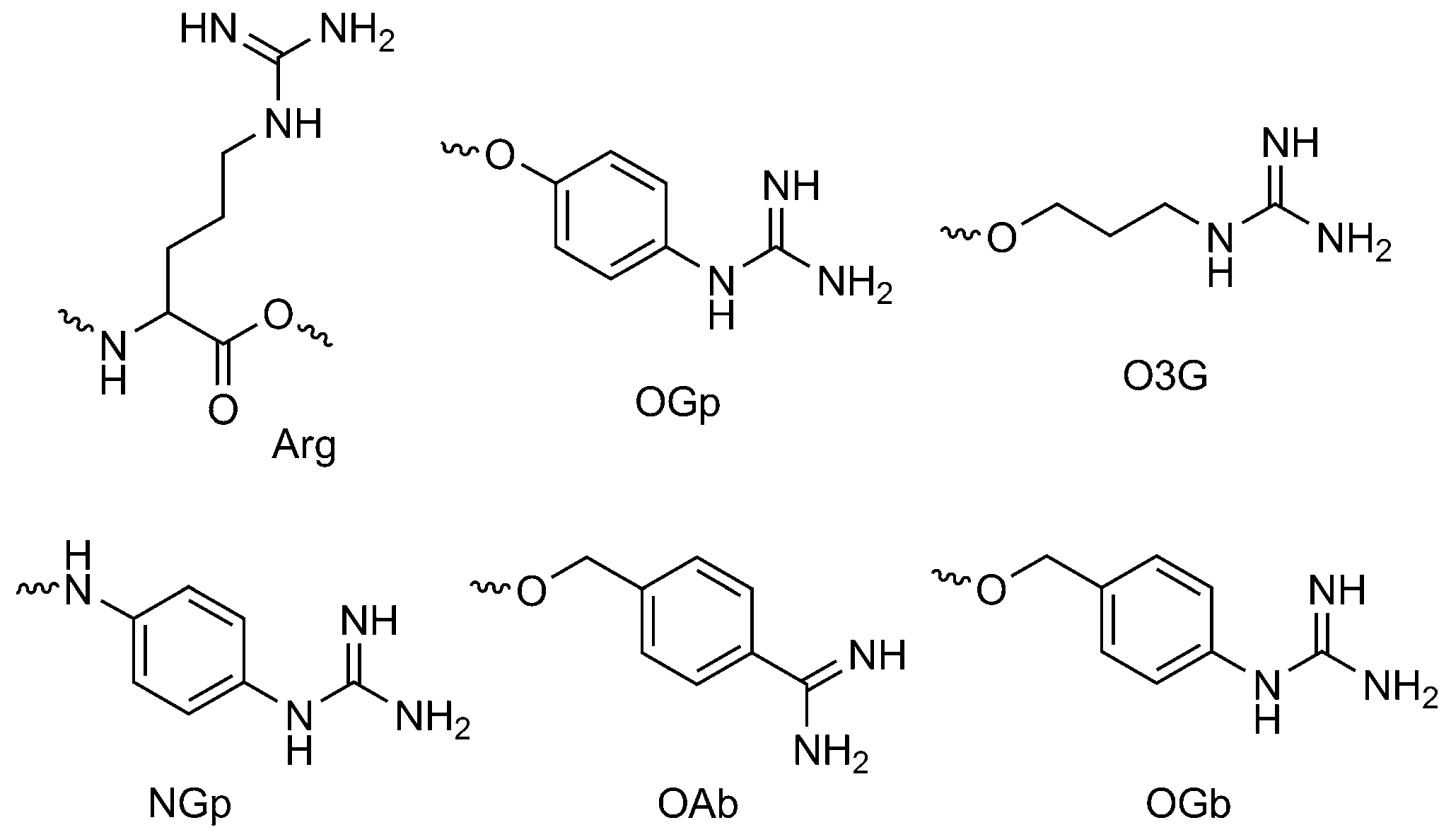
Structures of OGp analogues. OGp: *p*-guanidinophenyl ester; O3G: 3-guanidinopropyl ester; NGp: *p*-guanidinobenzyl amide; OAb: *p*-amidinobenzyl ester; OGb: *p*-guanidinobenzyl ester.

The analogues were designed in such a way that they closely resemble OGp in structure, although the leaving group character was varied considerably (Scheme 1).

Two benzylic variants (OAb and OGb) were synthesized as the corresponding Z-Gly-OH esters,[^5^] as well as an aliphatic analogue (O3G) and an OGp equivalent where the ester bond was replaced by an amide bond (NGp). The substrates were restricted to glycine esters in order to rule out any influence of the side chain in the coupling reactions. The anticipated analogy of these four compounds was evaluated by a computational docking study with the flexible docking program Fleksy.[^8^] The results were visualized and analyzed by using the YASARA program.[^9^]

The three-dimensional structure of trypsin has been previously solved by crystallographic studies.[^10^] The secondary structure of this globular enzyme consists of β-sheets organized into two densely packed hydrophobic β-barrels. Five subpockets (S_3_ through S_2_′)[^11^] are important for specific binding of the substrate. A crucial interaction occurs at the carboxylate moiety of Asp189 at the bottom of subpocket S_1_, which primarily determines the specificity of trypsin for positively charged side chains. The catalytic triad comprises Ser195, His57, and Asp102. The adjacent backbone amides of Gly193 and Ser195 create the oxyanion hole.

**Figure 1:**
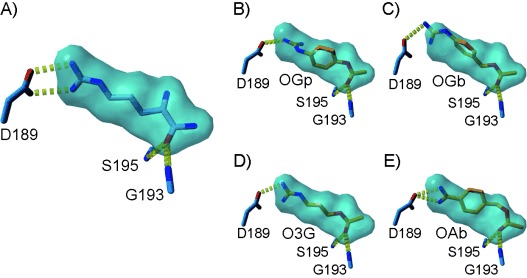
Molecular modeling of OGp analogues in trypsin. Hydrogen bonding interactions to functionally important amino acids in the trypsin active site are shown for A) arginine, B) OGp, C) OGb, D) O3G, and E) OAb. The space occupied by the arginine side chain is indicated in blue.

The Z-Gly-OGp ester (Figure 1 B) could be easily docked in the active site of trypsin, in a similar way to the arginine side chain (Figure 1 A), in agreement with previous findings.[^7^] The guanidino group made the equivalent crucial interaction with Asp189 in the S_1_ pocket and, additionally, formed hydrogen bonds with Ser190 and Trp215. Furthermore, the substrate carbonyl was nicely located in the oxyanion hole. The docking poses of the benzylic esters Z-Gly-OGb and Z-Gly-OAb were less optimal, even though the carbonyl groups were located in the oxyanion hole. Figure 1 C shows that OGb slightly extended beyond the volume occupied by the arginine side chain as a result of the additional carbon atom, whereas OAb (Figure 1 E) had to adopt a somewhat distorted conformation to be located in the oxyanion hole while simultaneously retaining interaction between the amidinium group and Asp189. The aliphatic Z-Gly-O3G analogue (Figure 1 D) perfectly mimicked the side chain of arginine, thus achieving hydrogen-bond interactions with Asp189, Ser190, and Tyr217. The oxyanion hole residues Gly193 and Ser195 were in the correct position to stabilize the carbonyl of the ester. Z-Gly-NGp showed a binding mode similar to that of Z-Gly-OGp.

### Experimentally determined activity of OGp analogues

The OGp analogues were evaluated experimentally in an enzymatic assay with trypsin. H–Phe–NH_2_ was used as the acyl acceptor because of its distinctive UV properties at 254 nm, which simplifies HPLC analysis. The enzymatic reaction was monitored for three hours. The identity of the products was confirmed by chemical synthesis of reference compounds and LC-MS analysis. Table 1 presents either the time to reach full conversion, or the extent of conversion after three hours. Background hydrolysis of the analogues was determined from a blank reaction (no trypsin). This indicated that the ratio of enzymatic synthesis to hydrolysis remained constant over time (measured at 24 h unless stated otherwise).

**Table 1:** Various Z-Gly-*Act* compounds tested experimentally.[a]

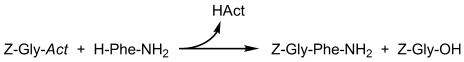						
		Experimental		Background[c]	Enzymatic	
	Act	*t* [min]	Conv [%]	Z-Gly-OH [%]	Z-Gly-Phe-NH_2_ [%]	Z-Gly-OH [%]
1	OGp[b]	15	100	2.7	22.5	74.8
2	OAb	180	43	2.0	23.4	17.6
3	OGb	120	99	3.3	72.5	23.2
4	NGp	180	–	–	–	–
5	O3G	180	38	0.9	29.6	7.5
6	O3G∇	180	73	5.0	54.0	14.0
7	O3G=	180	100	2.4	76.3	21.3
8	OTfe	90	100	5.7	73.6	20.7

[a] Conditions: 2 mm Z-Gly-*Act*, 15 mm Phe–NH_2_, 160 μm trypsin, 0.2 m HEPES (pH 8.0), 0.2 m NaCl, 20 mm CaCl_2_, 10 % (*v*/*v*) DMF.

[b] 1.6 μm trypsin, which is 100 times less than for the remaining entries.

[c] Only spontaneous hydrolysis was observed as background reaction.

Z-Gly-OGp is readily converted by trypsin, although the synthesis to hydrolysis ratio is not favorable (Table 1). The remaining analogues did not show any activity at the same enzyme concentration, except Z-Gly-OGb, for which there was slight activity. A 100-fold increase in trypsin concentration gave some differentiation: Z-Gly-OGb was almost completely consumed in two hours, whereas Z-Gly-OAb and Z-Gly-O3G were 43 and 38 % converted, respectively, in three hours. The lower activity with Z-Gly-OAb might be explained by its somewhat distorted fit in the active site of trypsin. The low activity with Z-Gly-O3G, however, was absolutely unexpected, as this analogue appeared to be a perfect mimetic. Z-Gly-NGp was the only analogue that remained completely inactive under these conditions. In all cases, the leaving group characteristics of the analogues were reduced compared to OGp. We conclude that although all the analogues were expected to be fairly similar in terms of affinity for the enzyme, this contribution to activity is rather small. Rather, the results imply a large influence of the leaving group ability of the ester.

### O3G variants with increased activity

If electronic properties indeed play an important role, it should be possible to increase the activity of the analogues by transforming them into better leaving groups. O3G was selected for this, as it appeared to be a near-perfect mimic of the natural substrate and its activity was surprisingly low compared to OGp.

**Figure 2:**
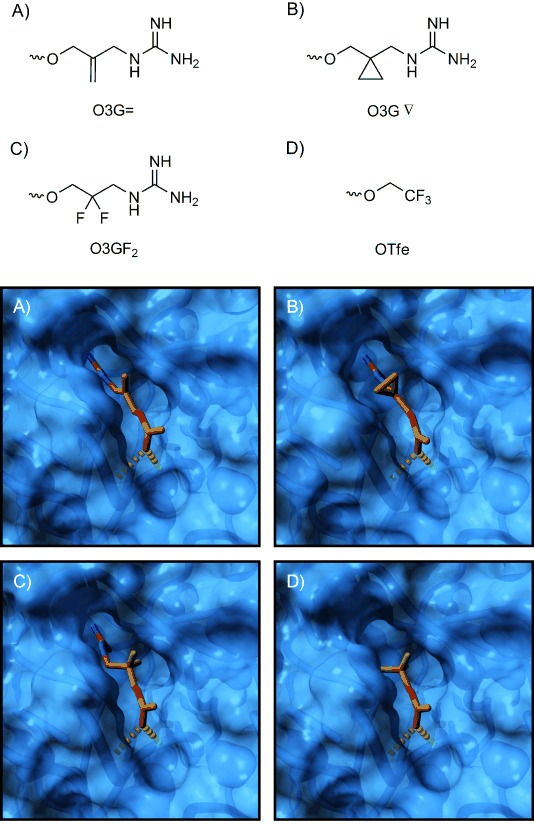
Structures of O3G variants and their docking poses. A) O3G=, B) O3G∇, C) O3GF_2_, and D) OTfe.

Inspection of the molecular model of trypsin with Z-Gly-O3G revealed that there is space in the binding pocket for the introduction of a small substituent (Figure 2), such as a methylene (O3G=) or two fluorides (O3GF_2_), which are weak and strong inductively electron-withdrawing groups, respectively. However, these modifications also create additional van der Waals interactions with the pocket, which by themselves can be a reason for increased affinity of the ester for the enzyme. To assess this effect, a cyclopropyl group was included (O3G∇), which was shown to fit in the binding pocket to make these additional interactions without altering the electronic properties.

The O3G variants were then experimentally evaluated in a trypsin enzymatic assay. As the synthesis of Z-Gly-O3GF_2_ failed (synthesis details are provided in the Supporting Information), the trifluoroethyl ester (OTfe) was included as an alternative, as it also nicely fits in the active site of trypsin (Figure 2 D) and it is known to act as a good leaving group. Both O3G variants showed considerably superior activity over O3G (Table 1). As anticipated, addition of the steric cyclopropyl group (O3G∇) increased the activity (from 38 to 73 % conversion in 3 h), whereas introduction of the slightly inductively electron-withdrawing methylene (O3G=) improved the activity even further (100 % conversion in 3 h). Surprisingly, of all the analogues tested, Z-Gly-OTfe yielded the fastest reaction (100 % conversion in 90 min), despite the absence of a cationic recognition element. This again supports the idea that the leaving group ability is an important contributor to the suitability of the ester for enzymatic peptide synthesis.

### Ab initio calculations

**Figure 3:**
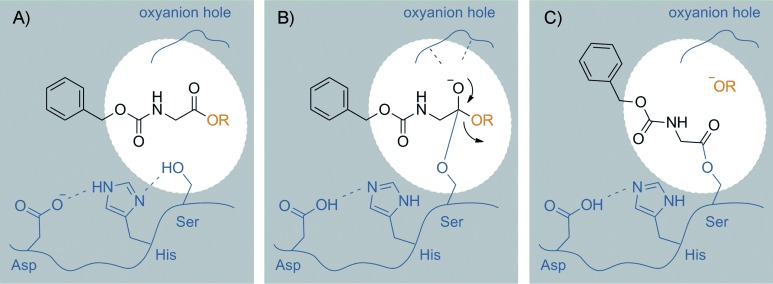
Reaction structural pathway of Z-Gly-Act with trypsin.

An appropriate computational technique to study electronic events within or between molecules is ab initio calculations. As the computational requirements are large for these calculations, four compounds were selected: Z-Gly-OGp, Z-Gly-O3G, Z-Gly-NGp, and Z-Gly-OTfe. Furthermore, the system was drastically simplified, in that the enzyme was represented by only the hydroxyl connected to a carbon of the active site serine. In addition, part of the protecting group of the ester was not taken into consideration. This situation is displayed as the white regions of Figure 3 A. The first step of the reaction comprises the formation of a tetrahedral intermediate, which is stabilized by the oxyanion hole (Figure 3 B). The subsequent collapse of the intermediate liberates the alcohol (or amine in the case of NGp) from the complex (Figure 3 C). This was the endpoint of our calculations, as we were interested in differences in leaving group ability. Obviously, the enzyme still needs to be deacylated by a nucleophile to complete the catalytic cycle, which would also proceed by a tetrahedral intermediate.

**Figure 4:**
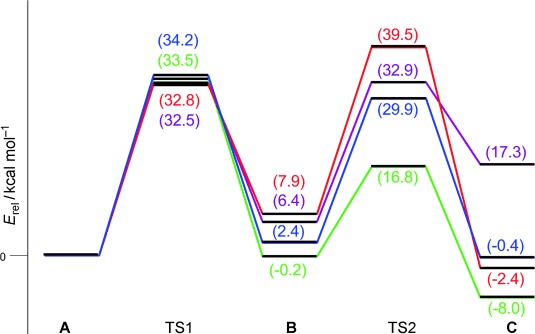
Energy diagram for R=OGp (green), O3G (purple), NGp (red), and OTfe (blue) derived from ab initio calculations. A, B and C correspond to the structures depicted in Table 2.

We set out to locate the tetrahedral intermediate for the OGp compound. After exhaustive partial optimizations (in which the C–O bond connecting OGp to the central carbon of the tetrahedral intermediate was fixed), we came to the conclusion that nowhere along this internal coordinate did a stationary point exist. No optimum or saddle point could be found. Removing the restraint of the fixed C–O bond always resulted in dissociation upon optimization. A stable intermediate was found, however, when a hydrogen atom was added to the oxygen carrying the negative charge. This is in line with the commonly accepted hypothesis[^12^] that, in the natural protein environment, hydrogen bonding in the oxyanion hole stabilizes the tetrahedral intermediate. As an equivalent amount of atoms and charge was required throughout the calculations, a hydrogen atom was added to the leaving group too. This corresponds with the accepted mechanism, in which the leaving group is protonated upon formation of the first tetrahedral intermediate.[^13^] With the stable intermediates identified, the energy diagram (Figure 4) and the data (Table 2) were produced.

**Table 2:** Ab initio computed energies.

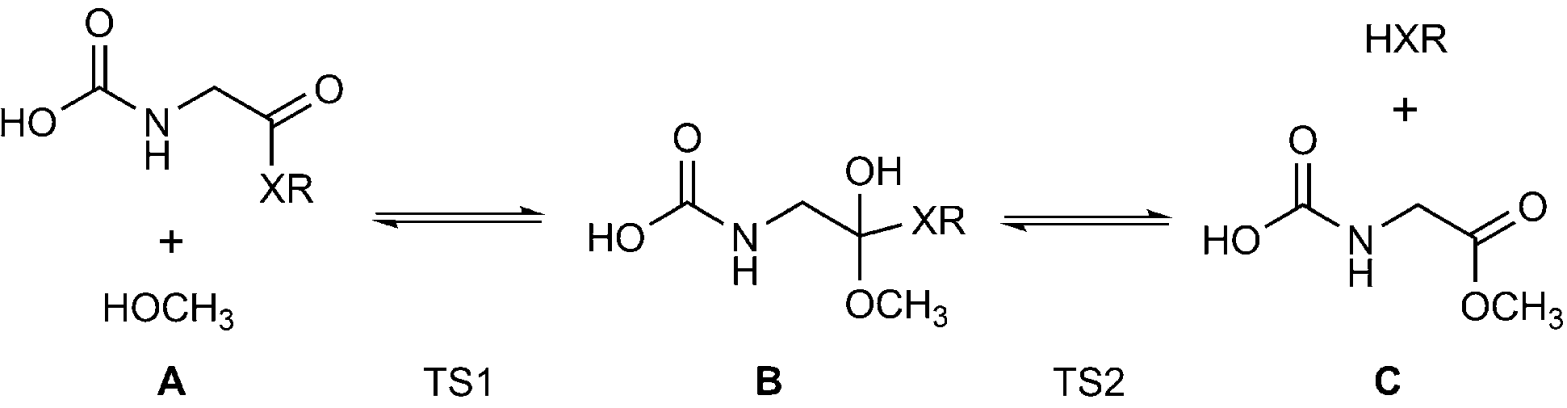				
Compound (XR)	Reaction coordinate[a]	E-B3LYP [Hartree]	ZPVE [Hartree]	*E* relative [kcal mol^−1^]
OGp	A	−1023.822519	0.318313	0
	TS (A⇒B)	−1023.768428	0.317477	33.5
	B	−1023.827721	0.323082	−0.2
	TS (B⇒C)	−1023.796406	0.318871	16.8
	C	−1023.836929	0.319910	−8.0
O3G	A	−910.799298	0.326420	0
	TS (A⇒B)	−910.745545	0.324387	32.5
	B	−910.793383	0.330697	6.4
	TS (B⇒C)	−910.744129	0.323695	32.9
	C	−910.770669	0.325304	17.3
NGp	A	−1003.978561	0.333122	0
	TS (A⇒B)	−1003.923725	0.330513	32.8
	B	−1003.970149	0.337313	7.9
	TS (B⇒C)	−1003.913334	0.330891	39.5
	C	−1003.981686	0.332390	−2.4
OTfe	A	−964.639842	0.194069	0
	TS (A⇒B)	−964.582210	0.193946	34.2
	B	−964.638998	0.200064	2.4
	TS (B⇒C)	−964.588930	0.193792	29.9
	C	−964.639257	0.195890	−0.4

[a] See Figure 4. TS, transition state.

In the diagram, the relative energies are compared; for convenience, the starting point for each of the reaction paths is arbitrarily set to zero. The activation energy (TS1) for the formation of tetrahedral intermediate (B) was similar for all compounds under investigation, although the energy level of B differed for each analogue. The next transition state (TS2), which is principally indicative of the leaving group ability of the various alcohols and amine, showed more variation but with a similar trend. The enzyme cooperatively stabilizes the negative charge that develops on the leaving group during the collapse of the tetrahedral intermediate (simulated by a proton). However, the differences between the leaving groups are mainly determined by the extent to which they can stabilize the developing negative charge. The low activation energy (TS2) of OGp reflects its ability to mesomerically stabilize the negative charge, whereas the high activation energy of NGp, also capable of mesomeric stabilisation, can be explained by the lower electronegativity of nitrogen compared to oxygen. Similarly, but to a lesser extent, the inductively electron-withdrawing effect of the OTfe group is helpful in stabilizing the negative charge. In the case of O3G, the negative charge is isolated on the oxygen, without possibilities for further stabilisation, which might also account for the high energy level at C.

In comparing these computational results with the experimental outcomes, one should bear in mind that only the acylation step was computationally studied. The experiments with trypsin provide insight in the efficiency of the complete catalytic cycle, including deacylation of the enzyme. In spite of this, the ranking OGp>OTfe>O3G>NGp from the ab initio calculations is in agreement with the experimentally determined activities of Z-Gly-OGp and its analogues. In addition, the marked contrast between the highly energetic tetrahedral intermediate and TS2 of NGp, and the favorable energies for OGp, seem to be consistent with the observation that acylation is the rate-determining step in amide hydrolysis,[^14^] in contrast to deacylation as rate-limiting step in OGp ester hydrolysis.[^15^] However, according to Menger et al. the reaction kinetics of proteases towards esters are highly dependent of the nature of the ester, as was demonstrated by comparing the *p*-nitrophenyl ester with the ethyl ester.[^16^] These authors argued that the “*p*-nitrophenyl ester syndrome” can be attributed to excellent electrophilic assistance to the departing entity. When reasoning by this analogy, it follows that the O3G ester must be inferior to OGp.

## Conclusions

Various methods were employed to determine the properties to which the success of the OGp ester as substrate mimetic and enzyme-specific activating ester can be attributed. Although a direct experimental approach to determine solely the affinity of a substrate is not available, a computational docking study of closely related OGp analogues was insightful.

Z-Gly-OGb and Z-Gly-OAb showed that a worse fit in the active site could be directly linked to a decrease in activity in the enzymatic assay. This was anticipated, as a good fit in the active site can be considered as an indication for affinity. To our complete surprise, however, it was also demonstrated that Z-Gly-O3G, although a perfect analogue of the natural substrate arginine according to docking studies, was barely active compared to Z-Gly-OGp. Apparently, recognition of the ester by the enzyme is alone insufficient for activity, in contrast to what has been suggested for substrate mimetics in the literature.

We hypothesized that a major contribution was the leaving group ability of the ester; this was supported by ab initio calculations that showed that OGp is a good leaving group whereas NGp and O3G are not. Furthermore, we were able to design improved O3G variants based on this hypothesis. Increasing activity was observed in the order O3G<O3G∇<O3G=<OTfe, the latter being the most effective because, although no cationic recognition element is present in this molecule, it is a good leaving group.

## Experimental Section

**Synthesis:** See the Supporting Information for a detailed description of the synthetic procedures and product characterization of the O3G variants. The details for the compounds in Scheme 1 can be found in a previous article.[^5^]

**Molecular modeling of trypsin–OGp analogue complexes:** All described molecular docking studies were performed by using the flexible docking program Fleksy.[^8^^,^^17^] The crystal structure of trypsin in complex with bovine pancreatic trypsin inhibitor (BPTI),[^18^] solved at 1.5 Å resolution, was used for the receptor structure (PDB ID: 3FP6). The structure was prepared for docking by removing BPTI and all water molecules, then hydrogen atoms were added and their positions were optimized by using the YASARA program.[^9^] In the applied docking protocol, only those docking poses in which the scissile bond of the docked substrate mimetic aligned to the scissile bond of the natural peptide substrate were taken forward. Otherwise, default parameters[^8^] were applied.

**General procedure for the enzymatic reactions:** Enzymatic acyl transfer reactions were performed at 25 °C in a total volume of 375 μL of HEPES (0.2 m, pH 8.0), NaCl (0.2 m), CaCl_2_ (20 mm), and DMF (10 %), with para toluene sulfonic acid (2 mm) as an internal standard. Stock solutions of Z-Gly-Act compounds (50 mm) in DMF and H-Phe-NH_2_ (30 mm) in buffer were prepared. The final concentrations of acyl donor and acyl acceptor were 2 mm and 15 mm, respectively. The latter was calculated as free, Nα-unprotonated nucleophile concentration, [HN]_0_, according to the Henderson–Hasselbalch equation: [HN]_0_=[N]_0_/(1+10 p*K*−pH). MilliQ water (1 mL, Millipore) was added to trypsin (9.6 mg), the solution was stirred, and aliquots were stored for a maximum of one month at −20 °C. Following thermal equilibration of the assay mixture, the enzymatic reaction was started by addition of trypsin (1.6 or 160 μm). Blank reactions were run in parallel (no trypsin). Spontaneous ester hydrolysis was determined from the control reaction, as well as nonenzymatic aminolysis of the acyl donor esters (the latter could be ruled out). At regular intervals, aliquots (20 μL) were withdrawn and quenched with glacial acetic acid (20 μL). The reactions were monitored for three hours by HPLC, and checked once more for changes in reaction mixture composition after 24 h. The values reported are the averages of at least two separate experiments. The identities of the formed peptide products were established by chemical synthesis of reference compounds and LC-MS.

**HPLC analysis:** Samples were analyzed by a LC 2010 analytical HPLC system (Shimadzu, Kyoto, Japan) equipped with a Varian Inertsil RP C18 column (ODS-3, 5 μm, 150×4.6 mm; Agilent) and eluted with various mixtures of acetonitrile/water containing trifluoroacetic acid (0.1 %) under isocratic and gradient conditions (flow rate 1.0 mL min^−1^, detection 254 nm). Product yields were calculated from peak areas of the substrate esters and the hydrolysis and aminolysis products.

**Ab initio calculations:** Standard LCAO-MO-SCF calculations were performed with the program Gamess-US[^19^] by employing restricted Hartree–Fock (RHF) procedures and density functional theory (DFT). All DFT calculations were carried out with the B3LYP exchange-correlation function.[^20^] The geometries of the isomers were determined by using analytical gradient and numerical second-derivative optimization procedures with the 6–31G** basis set. The relative energies were corrected for the contribution of zero-point vibrational energies (ZPVE). The ZPVEs were calculated for the 6–31G** optimized geometries by employing RHF and the 6–31G** basis set.
