# Supplementary material for: Enzyme-Specific Activation versus Leaving Group Ability
Source: Chembiochem. 2012 Jul 23;13(12):1785–90. doi: 10.1002/cbic.201200227 (PMC3569868; doi:10.1002/cbic.201200227)
Supplement: Supplementary file 1 [file cbic0013-1785-sd1.pdf]

CHEMBIOCHEM

Supporting Information

© Copyright Wiley-VCH Verlag GmbH & Co. KGaA, 69451 Weinheim, 2012

Enzyme-Specific Activation versus Leaving Group Ability

Roseri J. A. C. de Beer,[a] Berry Bögels,[a] Gijs Schaftenaar,[b] Barbara Zarzycka,[b]
Peter J. L. M. Quaedflieg,[c] Floris L. van Delft,[a] Sander B. Nabuurs,[b] and
Floris P. J. T. Rutjes*[a]

cbic_201200227_sm_miscellaneous_information.pdf

General remarks:

All chemicals were obtained from commercial sources and used without further purification, unless stated otherwise. If appropriate, reactions were carried out under an inert atmosphere of dry nitrogen or argon. Standard syringe techniques were applied for the transfer of dry solvents and air- or moisture-sensitive reagents. Reactions were followed and R_f values were obtained using thin layer chromatography (TLC) on silica gel-coated plates (Merck 60 F254) with the indicated solvent mixture. Detection was performed with UV-light, and/or by charring at ~150 °C after dipping into aqueous basic permanganate. Melting points were analysed with a Büchi melting point B-545. IR spectra were recorded on an ATI Mattson Genesis Series FTIR spectrometer, or a Bruker Tensor 27 FTIR spectrometer. NMR spectra were recorded on a Bruker DMX 300 (300 MHz and 75 MHz for ^{1}H and ^{13}C, respectively). ^{1}H-NMR chemical shifts are reported in parts per million (ppm) relative to tetramethylsilane (TMS) as internal standard, or a residual proton peak of the solvent: δ = 7.26 ppm for $CDCl_3$, δ = 3.31 ppm for CD_3OD, δ = 2.94 ppm for CD_3CN and δ = 2.50 ppm for DMSO-d_6. Multiplicities are reported as: s (singlet), d (doublet), t (triplet), q (quartet), dd (doublet of doublets), dt (doublet of triplets), dq (double quartet), ddd (double, double doublet), ddt (double, double triplet) or m (multiplet). Broad peaks are indicated by br. Coupling constants are reported as *J*-values in Hz. The number of protons (n) for a given resonance is indicated as nH, and is based on spectral integration values. ^{13}C-NMR chemical shifts (δ) are reported in ppm relative to $CDCl_3$ (δ = 77.0), CD_3OD (δ = 49.0), CD_3CN (δ = 1.24) or DMSO-d_6 (δ = 39.5). Column or flash chromatography was carried out using ACROS silica gel (0.035-0.070 mm, and ca 6 nm pore diameter). Optical rotations were determined with a Perkin Elmer 241 polarimeter. High resolution mass spectra were recorded on a JEOL AccuTOF (ESI), or a MAT900 (EI, CI, and ESI). Elemental analyses were carried out using a Carlo Erba Instruments CHNS-O EA 1108 element analyser.

General procedure: DCC-coupling of Cbz-protected amino acids with an alcohol

To a cooled solution (0 °C) of Z-X_{AA}-OH (1.4 equiv), alcohol (1 equiv) and DMAP (0.2 equiv) in EtOAc (10 mL), DCC (1.4 equiv) was added slowly. The reaction mixture was stirred at 0 °C for 1 h and an additional 2 h at room temperature. The solid DCU was filtered off before the solvent was evaporated *in vacuo.* In that case the reaction mixture was poured in citric acid (5%, 5 mL) and extracted with EtOAc (3 × 25 mL). The combined organic layers were washed with saturated $NaHCO_3$ (5 mL) and brine (10 mL), dried over sodium sulfate and then evaporated to dryness. The crude reaction mixture was purified with column chromatography.

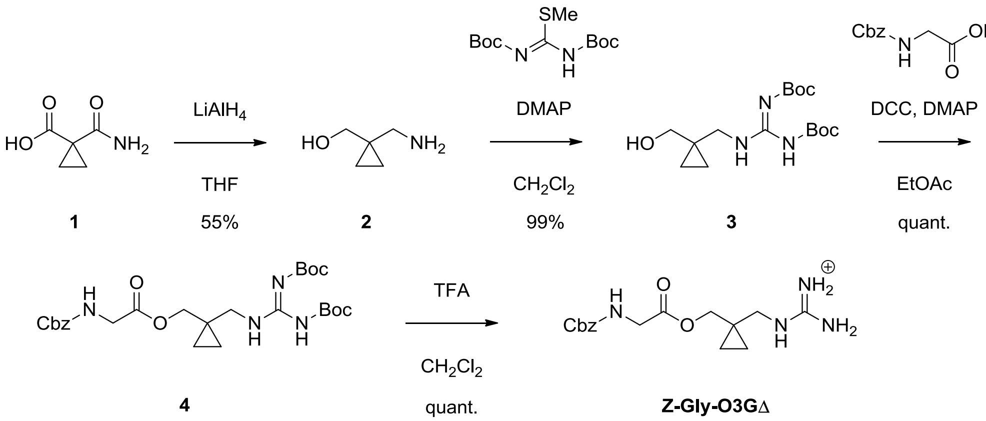

3-Amino-2-cyclopropylpropanol (2)

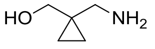

To a solution of $LiAlH_4$ (244 mg, 6.43 mmol, 6.4 equiv) in anhydrous THF (5 mL) containing 4 Å MS was added 1-(aminocarbonyl)cyclopropanecarboxylic acid (129 mg, 1.0 mmol, 1.0 equiv) and the mixture was refluxed overnight. Work-up was performed by evaporation of the solvent and slow addition of water (5 mL). Subsequently, THF (20 mL) was added and stirred for 30 minutes The resulting reaction mixture was evaporated *in vacuo*, and Et_2O (50 mL) was added to form a precipitate, which was filtered off. The residue was washed with Et_2O (3 × 50 mL) and the organic layers were combined, dried over Na_2SO_4, filtered, and evaporated *in vacuo* to afford **2** (74 mg, 55%) as a yellow oil. R_f 0.16 (10% MeOH in CH_2Cl_2). IR (film) 3356, 3280, 2996, 2920, 2865, 1653, 1598, 1425, 1023, 988, 933 cm^{-1}. ^{1}H NMR ($CDCl_3$, 300 MHz): δ 3.62 (s, 2H), 2.82 (s, 2H), 2.48 (br s, 2NH, 1OH), 0.51-0.46 (m, 2H), 0.42-0.37 (m, 2H). ^{13}C NMR ($CDCl_3$, 75 MHz): δ 68.2, 49.7, 30.9, 10.1, 9.8. HRMS (ESI) *m/z* calcd for $C_5H_{12}NO$ $(M+H)^+$: 102.0919, found: 102.0902.

3-[*N,N'*-di(Boc)guanidino]-2-cyclopropylpropanol (3)

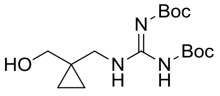

To a solution of *N,N'*-di(Boc)-*S*-methylisothiourea (290 mg, 1.0 mmol, 1.0 equiv) and DMAP (183 mg, 1.5 mmol, 1.5 equiv) in CH_2Cl_2 (5 mL) was added **2** (0.101 g, 1.0 mmol, 1.0 equiv) and stirred

at room temperature for 5 hours. The reaction mixture was evaporated to dryness and purified by means of column chromatography (MeOH in CH_2Cl_2, 0→5%) to afford **3** (341 mg, 99%) as a white fluffy solid. R_f 0.32 (5% MeOH in CH_2Cl_2). IR (film) 3328, 2968, 2920, 1722, 1660, 1612, 1556, 1425, 1369, 1314, 1238, 1162, 1134, 1058, 815, 753 cm^{-1}. ^{1}H NMR ($CDCl_3$, 300 MHz): δ 11.43 (br s, NH), 8.57 (br s, NH), 5.03 (t, *J* = 7.5 Hz, 1H, OH), 3.35-3.29 (m, 4H), 1.51 (s, 9H), 1.48 (s, 9H), 0.50-0.45 (m, 4H). ^{13}C NMR ($CDCl_3$, 75 MHz): δ 162.8, 157.0, 153.1, 83.5, 79.5, 66.0, 45.6, 30.9, 28.1, 23.4, 9.3. HRMS (ESI) *m/z* calcd for $C_{16}H_{30}N_3O_5$ $(M+H)^+$: 344.2186, found: 344.2172.

***N*$^{\alpha}$-Cbz-Glycine 3-[*N,N'*-di(Boc)guanidino]-2-cyclopropylpropyl ester (4)**

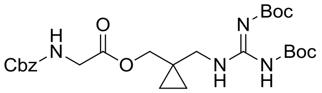

General procedure with adjusted amounts of every component using **3** (100 mg, 0.29 mmol, 1.0 equiv), DCC (72 mg, 0.35 mmol, 1.2 equiv), DMAP (43 mg, 0.35 mmol, 1.2 equiv) and Z-Gly-OH (73 mg, 0.35 mmol, 1.2 equiv) in CH_2Cl_2 (2 mL). The product (**4**) was obtained as a white solid after purification by column chromatography (MeOH in CH_2Cl_2, 0→5%) (155 mg, quant). R_f 0.44 (5% MeOH in CH_2Cl_2). IR (film) 3315, 2982, 1715, 1639, 1605, 1563, 1425, 1362, 1328, 1168, 1134, 1058, 815, 746, 691 cm^{-1}. ^{1}H NMR ($CDCl_3$, 300 MHz): δ 11.52 (s, 1H, NH), 8.59 (s, 1H, NH), 7.36-7.33 (m, 5H), 5.57 (s, 1H, NH), 5.13 (s, 2H), 4.09-4.06 (m, 4H), 3.41 (s, 2H), 1.49 (s, 18H), 0.88-0.62 (m, 4H). ^{13}C NMR ($CDCl_3$, 75 MHz): δ 206.8, 170.1, 163.4, 156.3, 156.0, 153.2, 136.3, 128.4, 128.2, 128.2, 128.1, 83.2, 71.4, 67.2, 66.9, 47.3, 43.1, 29.3, 18.9, 10.8. HRMS (ESI) *m/z* calcd for $C_{26}H_{39}N_4O_8$ $(M+H)^+$: 535.2768, found: 535.2747.

***N*$^{\alpha}$-Cbz-Glycine 3-guanidino-2-cyclopropylpropyl ester (Z-Gly-O3GΔ)**

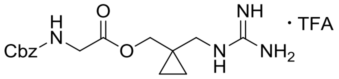

Compound **4** (65 mg, 0.12 mmol) was treated with CH_2Cl_2/TFA (2 mL, 1:1) overnight, the solvents were removed under reduced pressure and co-evaporated with ^{t}BuOH (3 × 10 mL). **Z-Gly-O3GΔ** (55 mg, quant) was obtained as a colourless oil. IR (film) 3349, 3169, 1674, 1535, 1258, 1189, 1134, 1037, 988, 836, 788, 760, 718, 712 cm^{-1}. ^{1}H NMR ($CDCl_3$, 300 MHz): δ 7.35-7.33 (m, 5H), 7.26 (br s, 2NH), 6.84 (br s, 3NH), 5.76 (br s, NH), 5.10 (s, 2H), 4.02 (s, 2H), 3.90 (s, 2H), 3.03 (s, 2H), 0.61-0.57 (m, 4H). ^{13}C NMR ($CDCl_3$, 75 MHz): δ 170.0, 157.5, 157.2, 135.8, 128.6, 128.4, 127.8, 70.1, 67.5, 46.9, 43.1, 31.0, 19.4, 9.8. HRMS (ESI) *m/z* calcd for $C_{16}H_{23}N_4O_4$ $(M+H)^+$: 335.1719, found: 335.1713.

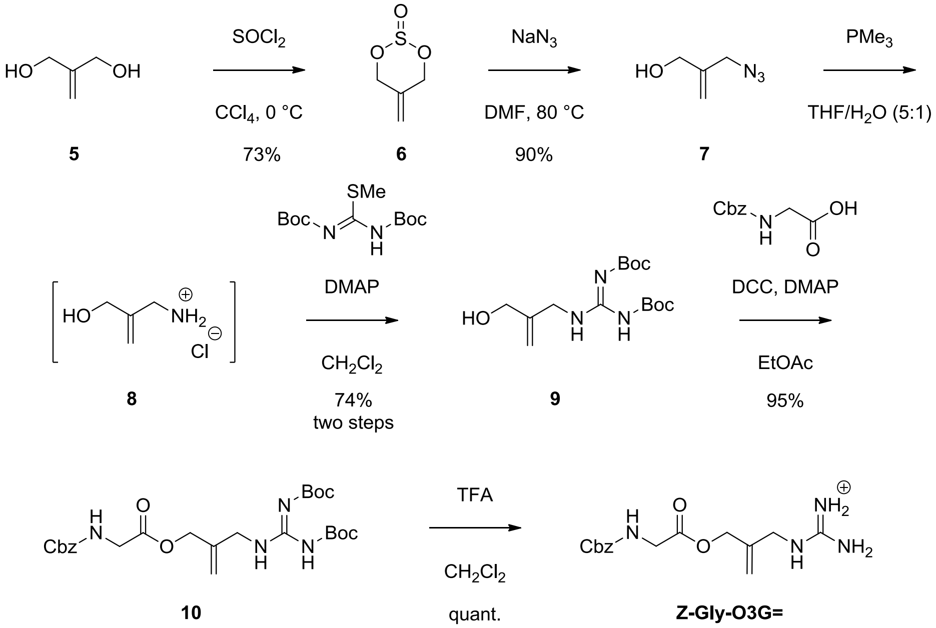

5-Methylene-2-oxo[1,3,2]dioxathiane (6)

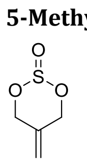

To an emulsion of diol 2-methylene-1,3-propanediol (1.76 g, 20 mmol, 1.0 equiv) in CCl_4 (12 mL) at 0 °C was added dropwise a solution of thionyl chloride (3.53 g, 30 mmol, 1.5 equiv) in CCl_4 (6 mL) under vigorous stirring. When the evolution of HCl had ceased after approximately 30 minutes, the solution was stirred for an additional 15 minutes Evaporation of the solvent at 0 °C and 5 mbar, followed by Kugelrohr distillation (b.p. 90-110 °C/ >10 Torr) yielded **6** (1.95 g, 73 % yield) as a colourless liquid. R_f 0.55 (EtOAc/pentane 1:4). IR (film) 3088, 2991, 2938, 2873, 1461, 1446, 1416, 1342, 1300, 1239, 1195, 1177, 982, 958, 926, 870, 759, 717, 692, 663 cm^{-1}. ^{1}H NMR ($CDCl_3$, 300 MHz): δ 5.35 (dt, *J* = 1.5, 13.2 Hz, 2H), 5.13-5.11 (m, 2H), 4.24 (dt, *J* =1.5, 13.2 Hz, 2H). ^{13}C NMR ($CDCl_3$, 75 MHz): δ 135.5, 114.4, 61.6. Spectral data were in accordance with those reported in literature.[7]

3-Azido-2-methylenepropanol (7)

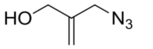

To a solution of **6** (630 mg, 4.7 mmol, 1.0 equiv) in DMF (10 mL) was added sodium azide (366 mg, 5.6 mmol, 1.2 equiv) and the mixture was heated to 80 °C. After 30 minutes, the reaction was quenched by addition of water (20 mL) and extracted with Et_2O (3 × 10 mL). The combined organic extracts were washed with water (20 mL), dried with $MgSO_4$ and concentrated *in vacuo* at 0 °C and 5 mbar. The crude product was purified by column chromatography (EtOAc/pentane 1:4) to obtain **7** (478 mg, 90%) as a yellow oil. R_f 0.24 (EtOAc/pentane 1:4). IR (film) 3308, 2913, 2858, 2089, 1653, 1445, 1238, 1072, 1016, 919, 871, 656, 559 cm^{-1}. ^{1}H NMR ($CDCl_3$, 300 MHz): δ 5.28 (s, 1H), 5.18 (s, 1H), 4.19 (s, 2H), 3.86 (s, 2H), 1.72 (s, 2H). ^{13}C NMR ($CDCl_3$, 75 MHz): δ 142.7, 114.6, 64.0, 53.3. Spectral data were in accordance with those reported in literature.[7]

3-[*N,N'*-di(Boc)guanidino]-2-methylenepropanol (9)

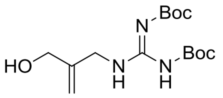

To a solution of **7** (113 mg, 1.0 mmol, 1.1 equiv) in water/THF (30 mL, 1:5) was added trimethylphosphine (84 mg, 1.1 mmol, 1.1 equiv) and the resulting mixture was stirred at room temperature overnight. Subsequently, HCl (1.5 mL, 1.0 M) was added and the solvent was evaporated *in vacuo* to afford crude **8** as a yellow oil. Without further purification crude **8** was added to a solution of *N,N'*-di(Boc)-*S*-methylisothiourea (145 mg, 0.50 mmol) and DMAP (92 mg, 0.75 mmol) in CH_2Cl_2 (5 mL) and the mixture was stirred at room temperature for 5 h. After evaporation to dryness and purification by means of column chromatography (MeOH in CH_2Cl_2, 0→5%) **9** was obtained as a white fluffy solid (242 mg, 74% over two steps). R_f 0.63 (5% MeOH in CH_2Cl_2). IR (film) 3324, 2952, 2927, 1729, 1653, 1612, 1432, 1301, 1245, 1167, 1127, 1085, 760, 680, 615 cm^{-1}. ^{1}H NMR ($CDCl_3$, 300 MHz): δ 11.43 (br s, NH), 8.57 (br s, NH), 5.05 (s, 1H), 4.99 (s, 1H), 4.87 (br s, OH), 4.08-4.04 (m, 4H), 1.50 (s, 9H), 1.48 (s, 9H). ^{13}C NMR ($CDCl_3$, 75 MHz): δ 162.6, 156.7, 153.1, 145.8, 114.0, 83.6, 79.6, 63.4, 43.0, 28.2, 28.0.

***N*^{α}-Cbz-Glycine 3-[*N,N'*-di(Boc)guanidino]-2-methylenepropyl ester (10)**

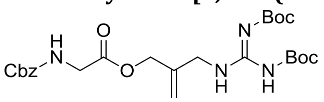

General procedure with adjusted amounts of every component using **9** (120 mg, 0.40 mmol, 1.0 equiv), DCC (83 mg, 0.40 mmol, 1.0 equiv), DMAP (49 mg, 0.40 mmol, 1.0 equiv) and Z-Gly-OH (84 mg, 0.40 mmol, 1.0 equiv) in EtOAc (2 mL). Purification was performed by column chromatography (EtOAc/heptane 1:3) to afford **10** as a white solid (181 mg, 95%). R_f 0.41 (EtOAc/heptane 1:1). IR (film) 3328, 2982, 2927, 1729, 1653, 1612, 1411, 1369, 1321, 1245, 1168, 1127, 1092, 1072, 822, 760, 691, 615 cm^{-1}. ^{1}H NMR ($CDCl_3$, 300 MHz): δ 11.52 (br s, NH), 8.48 (br s, NH), 7.41-7.32 (m, 5H), 5.45 (br s, NH), 5.24 (s, 2H), 5.13 (s, 2H), 4.66 (s, 2H), 4.16-4.10 (m, 2H), 4.09-4.00 (m, 2H), 1.49 (s, 18H). ^{13}C NMR ($CDCl_3$, 75 MHz): δ 163.4, 156.2, 153.3, 139.1, 136.3, 128.5, 128.2, 128.1, 117.1, 83.4, 79.5, 67.0, 66.4, 43.2, 42.9, 34.0, 28.2, 28.1, 24.9. HRMS (ESI) *m/z* calcd for $C_{25}H_{37}N_4O_8$ $(M+H)^+$: 521.2611, found: 521.2592.

***N*^{α}-Cbz-Glycine 3-guanidino-2-methylenepropyl ester (Z-Gly-O3G=)**

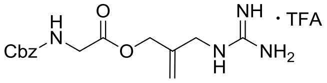

Compound **10** (100 mg, 0.20 mmol) was treated with CH_2Cl_2/TFA (6 mL, 1:1) overnight, the solvents were removed under reduced pressure and co-evaporated with ^{t}BuOH (3 × 10 mL). **Z-Gly-O3G=** was obtained as a colourless oil (83 mg, quant). IR (film) 3356, 3190, 2941, 1667, 1528, 1452, 1272, 1182, 1044, 975, 836, 795, 732, 691 cm^{-1}. ^{1}H NMR ($CDCl_3$, 300 MHz): δ 7.67 (br s, NH), 7.34-7.32 (m, 5H), 6.91 (br s, 2NH), 5.59 (br s, NH), 5.33 (d, J = 11.1 Hz, 2H), 5.10 (s, 2H), 4.67 (s, 2H), 3.92 (d, J = 6.0 Hz, 2H), 3.77 (d, J = 6.3 Hz, 2H), 3.38 (br s, 2H). ^{13}C NMR ($CDCl_3$, 75 MHz): δ 169.7, 157.6, 137.8, 135.8, 128.6, 128.4, 127.8, 118.9, 67.5, 66.0, 43.9, 43.0, 33.2, 31.2. HRMS (ESI) *m/z* calcd for $C_{15}H_{21}N_4O_4$ $(M+H)^+$: 321.1563, found: 321.1555.

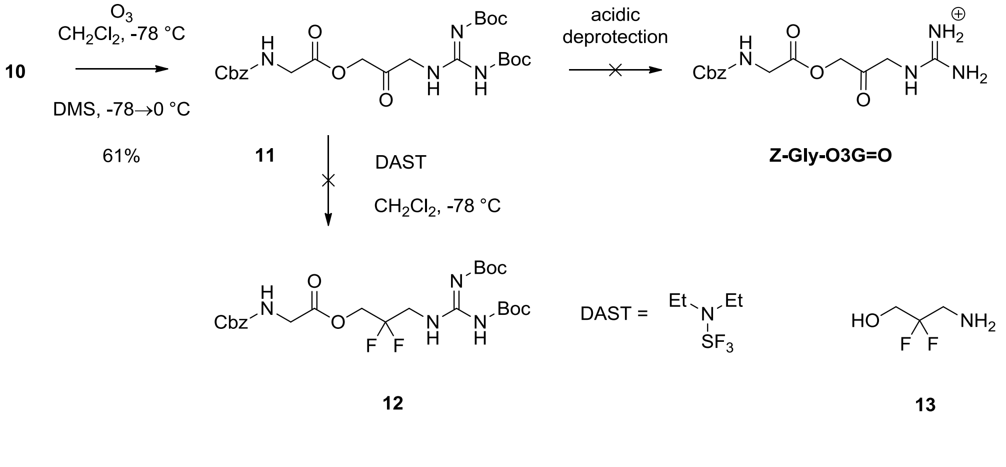

The synthesis of **Z-Gly-O3G$_{F2}$** was attempted by utilising DAST as nucleophilic fluoride donor on ketone **10** (Scheme above). Unfortunately, no reaction occurred. Alternative approaches, involving the synthesis of fluorinated amino alcohol **13** by various methods, failed to succeed.

N^{α}-Cbz-Glycine-3-[*N,N'*-di(Boc)guanidino]-2-carbonylpropyl ester (11)

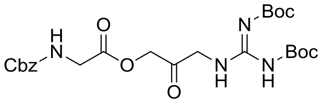

A solution of **10** (260 mg, 0.50 mmol, 1.0 equiv) in CH_2Cl_2 (10 mL) was stirred and cooled to −78 °C. Subsequently, ozone was bubbled through the solution for 30 minutes. The solution turned light blue and TLC-analysis indicated the disappearence of the substrate. Then DMS (34 mg, 0.55 mmol, 1.1 equiv) was added in one portion, stirring still continued at −78 °C for 10 minutes. The reaction mixture was brought to room temperature and stirred for an additional 10 minutes. Then the reaction was quenched with water (10 mL), the layers were separated and the waterlayer was washed with CH_2Cl_2 (3 × 5 mL). The combined organic layers were dried with Na_2SO_4 and the solvent was evaporated *in vacuo*. Purification was performed by means of column chromatography (EtOAc/heptane 1:4) to afford **11** (159 mg, 61%). R_f 0.50 (EtOAc/heptane 1:1). IR (film) 3322, 2975, 2920, 1715, 1646, 1618, 1563, 1411, 1355, 1300, 1155, 1065, 753 cm^{-1}. ^{1}H NMR ($CDCl_3$, 300 MHz): δ 11.39 (br s, NH), 8.96 (br s, NH), 7.36-7.32 (m, 5H), 5.52 (br s, NH), 5.13 (s, 2H), 4.81 (s, 2H), 4.36 (s, 2H), 4.12 (d, J = 5.7 Hz, 2H), 1.50 (s, 18H). ^{13}C NMR ($CDCl_3$, 75 MHz): δ 209.1, 170.4, 157.6, 136.8, 136.4, 128.6, 128.4, 127.8, 115.9, 67.5, 67.1, 66.0, 42.8, 30.1.
